# Acetate of Lead in Epistaxis

**Published:** 1851-12

**Authors:** D. H. Agnew

**Affiliations:** Philadelphia


### *Acetate of Lead in Epistaxis.* By D. H. Agnew, M. D., of Philadelphia.

During the month of November, 1850, I was called to see Mr. N., who, with the exception of a very short interval, had been bleeding from the nose for four hours. No probable cause could be ascribed for the attack. When I saw him first, the blood was flowing quite rapidly from the nostrils, showing no disposition to coagulate. He complained of a sense of tightness across the forehead (not amounting to pain.) The pulse, though small and quick, possessed a degree of firmness, which, taken together with other symptoms, induced me to believe the discharge to be dependent upon some temporary congestion of the vessels of the head. In accordance with this view of the case, the arm was tied up, and a quantity of blood, sufficiently large to make a decided impression upon the general system, abstracted; the head and shoulders elevated; cold applications to the former; a warm stimulating pediluvium, with directions to draw into the nostrils occasionally a solution of sulphate of zinc, and alum. This treatment, though it had the effect of diminishing for a short time the hemorrhage, failed entirely in arresting it, and before the lapse of an hour, the flow became as great as at the commencement of the attack. The patient now began to feel the loss of blood very much, the pulse failed, the face became pallid, and the nervous system excessively agitated. It was now proposed to plug the nostrils, preparatory to any other measures for relief, but to this the patient obstinately objected. At this time the acetate of lead was suggested, from its known efficacy in controlling hemorrhages from other parts. Ten grains were immediately administered in a little acetic acid, with directions to take a second powder, containing five grains, two hours and a half subsequently. Before the period came round for taking the second dose, the discharge had materially diminished, notwithstanding which it was given; and an hour following, it had entirely subsided. The rationale of its operation seemed in this case to be beautifully and strikingly exemplified. The first effect was its sedative influence over the circulating system, apparent by the rapid diminution in the frequency of the pulse. Second, its sympathetic action, (as I believe,) through the medium of the nervous system upon the calibre of the capillary vessels of the Schneiderian membrane lining the nasal cavities, obvious in the diminution of the flow. And, lastly, its effect upon the sanguine fluid itself, by increasing its coagulability. As a remedy in the different forms of profluvia, its value is well known to the profession; yet we think more decided results would be obtained, were it administered in much larger doses than are usually recommended. I would not encourage the idea that caution in its use is unnecessary, though I do think the danger from its free employment greatly exaggerated. While writing, a case of uterine hemorrhage occurs to me, where a fifteen grains dose of the acetate of lead effectually arrested the discharge, and saved the patient's life, after the failure of every other resource, the lead among the rest, though exhibited in small doses. The precautionary measure of administering the article with acetic acid, has not been neglected; and perhaps it is by thus preventing its conversion into the carbonate, that no ill results have been witnessed. Seeing no similar therapeutical application of this agent, it is reported, in order that it may receive a further trial.

